# Revitalizing the ethanologenic bacterium *Zymomonas mobilis* for sugar reduction in high-sugar-content fruits and commercial products

**DOI:** 10.1186/s40643-021-00467-2

**Published:** 2021-12-02

**Authors:** Mimi Hu, Xiangyu Chen, Ju Huang, Jun Du, Mian Li, Shihui Yang

**Affiliations:** 1 State Key Laboratory of Biocatalysis and Enzyme Engineering, Environmental Microbial Technology Center of Hubei Province, and School of Life Sciences, Hubei University, Wuhan, 430062 China; 2 China Biotech Fermentation Industry Association, Beijing, 100833 China; 3 Zhejiang Huakang Pharmaceutical Co., Ltd., Kaihua County, Zhejiang, China

**Keywords:** *Zymomonas mobilis*, *Saccharomyces cerevisiae*, Fruits, Chinese wine, Fermentation, Sugar reduction

## Abstract

The excessive consumption of sugars can cause health issues. Different strategies have been developed to reduce sugars in the diets. However, sugars in fruits and commercial products may be difficult to reduce, limiting their usage among certain populations of people. *Zymomonas mobilis* is a generally recognized as safe (GRAS) probiotic bacterium with the capability to produce levan-type prebiotics, and thrives in high-sugar environments with unique characteristics to be developed for lignocellulosic biofuel and biochemical production. In this study, the sugar reduction capabilities of *Z. mobilis* ZM4 were examined using two fruits of pear and persimmon and three high-sugar-content commercial products of two pear pastes (PPs) and one Chinese traditional wine (CTW). Our results demonstrated that *Z. mobilis* ZM4 can utilize sugars in fruits with about 20 g/L ethanol and less than 5 g/L sorbitol produced within 22 h using pears, and about 45 g/L ethanol and 30 g/L sorbitol produced within 34 h using persimmons. When PPs made from pears were used, *Z. mobilis* can utilize nearly all glucose (ca. 60 g/L) and most fructose (110 g/L) within 100 h with 40 ~ 60 g/L ethanol and more than 20 g/L sorbitol produced resulting in a final sorbitol concentration above 80 g/L. In the high-sugar-content alcoholic Chinese traditional wine, which contains mostly glucose and ethanol, *Z. mobilis* can reduce nearly all sugars with about 30 g/L ethanol produced, resulting in a final ethanol above 90 g/L. The ethanol yield and percentage yield of *Z. mobilis* in 50 ~ 60% CTW were 0.44 ~ 0.50 g/g and 86 ~ 97%, respectively, which are close to its theoretical yields—especially in 60% CTW. Although the ethanol yield and percentage yield in PPs were lower than those in CTW, they were similar to those in fruits of pears and persimmons with an ethanol yield around 0.30 ~ 0.37 g/g and ethanol percentage yield around 60 ~ 72%, which could be due to the formation of sorbitol and/or levan in the presence of both glucose and fructose. Our study also compared the fermentation performance of the classical ethanologenic yeast *Saccharomyces cerevisiae* BY4743 to *Z. mobilis*, with results suggesting that *Z. mobilis* ZM4 had better performance than that of yeast *S. cerevisiae* BY4743 given a higher sugar conversion rate and ethanol yield for sugar reduction. This work thus laid a foundation for utilizing the advantages of *Z. mobilis* in the food industry to reduce sugar concentrations or potentially produce alcoholic prebiotic beverages.

**Graphical Abstract:**

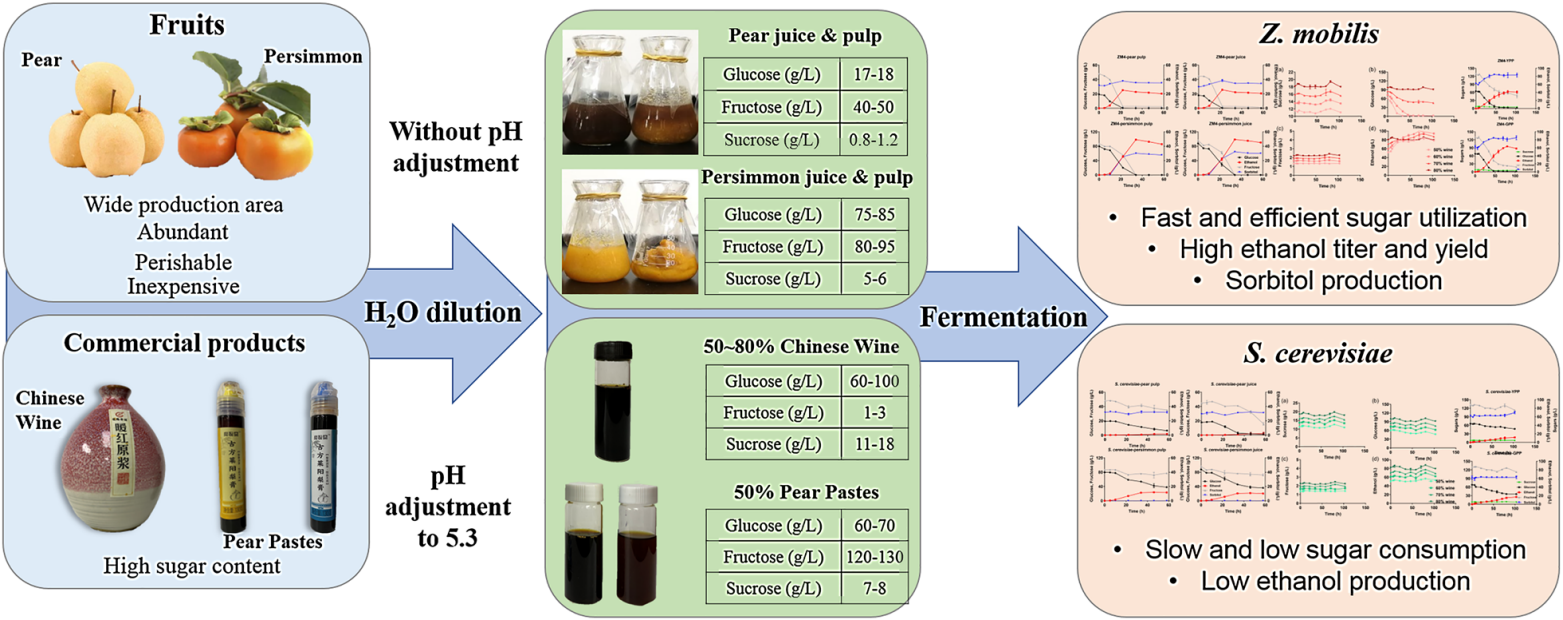

**Supplementary Information:**

The online version contains supplementary material available at 10.1186/s40643-021-00467-2.

## Introduction

Consumption of high-sugar content drinks and products affect the absorption of nutrients such as protein and vitamins, and increase the risk of kidney stones, obesity, diabetes, cardiovascular disease, oral diseases, and even cancers (Bantle et al. 2009; Delli Bovi et al. 2017; Febbraio et al. 2021; Johnson et al. 2018; Kohn et al. 2017; Taylor et al. 2021). To promote development of the low-sugar-content industry, many countries and regions have propagated and promoted sugar reduction to be under mandatory national control, and have also gradually formulated and promulgated the taxation policy for high-sugar-content foods. Besides controlling the diet and pharmacotherapy treatment (Apovian and Gokce 2012), different strategies have been developing to address the dilemma associated with the popularity of consuming high-sugar-content products and the pursuit of a healthy lifestyle with the usage of sugar substitutes such as sugar alcohols and artificial sweeteners. Common sugar alcohols include xylitol, erythritol, and sorbitol that can be derived from sugars, but have lower caloric content than sugars.

Sugars in fruits and some commercial high-sugar-content products in some cases are difficult and/or complicated to be reduced or replaced with sugar substitute due to the manufacturing techniques and processes used. The classical industrial ethanologen *Saccharomyces cerevisiae* is usually used for fruit wine production. For example, *S. cerevisiae* SY was used to ferment Dangshan pear with 14.10 ± 0.27% (v/v) ethanol produced from an initial 240 g/L total sugar within 15 days (Yang et al. 2019). Yeast was also used to ferment banana (Idise and Odum 2011), and pomegranate juice with 10.91 ± 0.27% (v/v) ethanol produced under a lower temperature less than 22 ℃ and a long fermentation time (Berenguer et al., 2016). Other microorganisms were also used with yeast to ferment sugars in the fruits. For example, *S. cerevisiae* and *Acetobacter aceti* were used to ferment waste pineapple residues for the production of fruit wine and vinegar in two consecutive steps for approximately 40 days with a final 7% (v/v) ethanol and 5% (v/v) acetic acid obtained (Roda et al. 2017).

*Zymomonas mobilis* is another model ethanologen, which has been traditionally used to make the alcoholic beverage “Pulque” in Central Mexico from the fermented sap of the agave plant for more than one thousand years. *Zymomonas* sp. was also isolated in juices from fruits and plants, such as cider and ale (Millis 1956). Due to its unique physiological characteristics and ideal industrial biocatalyst properties such as high sugar uptake and utilization efficiency, high osmolarity and ethanol tolerance, and high ethanol yield, significant efforts have been made to further understand and engineer *Zymomonas* as a robust microbial cell factory for lignocellulosic bioproducts. Many omics studies have already been performed and genetic engineering techniques developed, such as different CRISPR–Cas techniques (Jacobson et al. 2019; Jones-Burrage et al. 2019; Liu et al. 2020; Martien et al. 2019; Ong et al. 2020; Shen et al. 2019; Stoneman et al. 2020; Tatli et al. 2019; Vera et al. 2020; Zheng et al. 2019).

Although *Z. mobilis* could be an ideal host for lignocellulosic bioproducts, it is disadvantageous for bioethanol production using grains since it does not have enzymes such as amylase and maltase to utilize sugars other than sucrose, glucose, and fructose (Xia et al. 2019). It seems that the high sugar environment of fruit saps that *Z. mobilis* evolved to thrive in shaped its capability of utilizing sugars of sucrose, glucose, and fructose that usually exist in fruits, and the unique features of its hopanoid membrane structure and anaerobic Entner–Doudoroff (ED) pathway with efficient enzymes of pyruvate decarboxylase (Pdc) and alcohol dehydrogenases (Adhs) help it tolerate and efficiently utilize high concentration sugars for high ethanol production and tolerance (Brenac et al. 2019; Felczak et al. 2021; Todhanakasem et al. 2020; Wang et al. 2018; Yang et al. 2021) (Additional file 1: Fig. S1).

Moreover, *Z. mobilis* can produce levan by levansucrase SacB when sucrose is present in the media or produce sorbitol by glucose-fructose oxidoreductase (Gfo, EC 1.1.1.99) when either sucrose or both fructose and glucose are present is used (Jonas and Silveira 2004; Liu et al. 2010; Silbir et al. 2014; Tastan et al. 2019) (Additional file 1: Fig. S1). Although it is not economic to use *Z. mobilis* for bioethanol production using grains due to the formation of sorbitol and levan, which significantly compromise ethanol yield, it could be advantageous for applying *Z. mobilis* in food industry. Sorbitol is used in the food industry as a sweetener, humectant, and softener (Rice et al. 2020; Silveira and Jonas 2002), which can be found in many fruits, such as berries, pears, and apples (Jonas and Silveira 2004). The formation of sorbitol could also provide *Z. mobilis* protection under high osmotic environments such as the high sugar and ethanol conditions (Loos et al. 1994; Parker et al. 1997).

Compared with yeast, *Z. mobilis* metabolizes glucose faster and produces ethanol more efficiently than *S. cerevisiae* with a higher ethanol yield due to its unique anaerobic ED pathway and efficient Pdc and Adh enzymes resulting in less ATP and biomass produced for more sugar to be used in ethanol production (Todhanakasem et al. 2020; Yang et al. 2016, 2021). In addition, as a Gram-negative facultative anaerobic bacterium, *Z. mobilis* does not need oxygen control during fermentation, which can help simplify the fermentation processing and reduce infrastructure investment and fermentation cost.

Despite the excellent features discussed above, the intrinsic capability that *Z. mobilis* has to efficiently consume sugars of sucrose, glucose, and fructose in high sugar environments for high ethanol production as a microbial biocatalyst has not been fully explored and applied in the food industry—especially the sugar reduction of high-sugar-content fruits and commercial products (Aziz 2011; Musatti et al. 2018). We chose several high-sugar-content fruits and commercial products that are typical and popular in China to investigate the sugar reduction capability of *Z. mobilis* in these materials.

In this study, we evaluated and compared the performance of two ethanologens of *Z. mobilis* and *S. cerevisiae* to ferment sugars in two common fruits being pear and persimmon as well as three high-sugar-content commercial products being two traditional pear pastes and one Chinese traditional wine. Our work demonstrated that *Z. mobilis*, a fascinating probiotic bacterial ethanologen with the capability to produce sugar substitute of sorbitol and levan-type prebiotics, is an ideal microorganism for sugar reduction and sugar-free prebiotic beverages and products.

## Materials and methods

### Preparation of fermentation media using fruits and high-sugar products

Seasonal fresh fruits of Hebei Snow Pears and Guangxi persimmons were purchased from local grocery stores in Wuhan, China. The reason for selecting these two fruits is because of their availability, cost, and sugar content. Persimmon production in China is abundant, accounting for 43% of the world’s production in 2013 (Zou et al. 2017). Although ripe persimmon fruits are full of nutrients such as protein, vitamins, minerals, and dietary fibers (Hwang et al. 2017; Zhu et al. 2014), persimmons have thin skin and fast ripening period, resulting in a short shelf life (Hidalgo et al. 2012). Therefore, diverse and efficient strategies are needed to fully utilize these fruits before they are spoiled.

High-sugar-content commercial products of yellow pear paste (YPP) and green pear paste (GPP), cough syrups made of pears used in Traditional Chinese Medicine, were supplied by LingHang Food Company (Shandong, China). The high-sugar-content alcoholic product of Chinese traditional wine (CTW) was provided by WenTianGe Biological Company (Shandong, China).

Clean and dry pears and persimmons were directly diced into small pieces without removing fruit peels to generate a fruit slurry. Half of the fruit slurry was directly used as fruit pulp for fermentation after adding distilled water (dH_2_O), and the other half was filtered through filter paper to remove solid materials in the slurry as fruit juice. Commercial products of YPP, GPP, and CTW were also diluted using dH_2_O (Table 1).

**Table 1:** Recipe of fermentation media used in this study and initial concentrations of sugars and sorbitol in the media

Materials	Recipe	Initial concentration (g/L)				
		Sucrose	Glucose	Fructose	Ethanol	Sorbitol
50% CTW	50 mL CTW, 50 mL dH_2_O	11.54 ± 0.05	61.95 ± 0.01	1.16 ± 0.33	51.68 ± 0.13	0
60% CTW	60 mL CTW, 40 mL dH_2_O	13.58 ± 0.16	72.52 ± 0.73	1.64 ± 0.02	59.66 ± 0.90	0
70% CTW	70 mL CTW, 30 mL dH_2_O	15.89 ± 0.03	84.97 ± 0.05	1.95 ± 0.06	70.87 ± 0.13	0
80% CTW	80 mL CTW, 20 mL dH_2_O	18.12 ± 0.07	96.75 ± 0.49	2.17 ± 0.01	80.08 ± 0.24	0
YPP	130 g pear paste,	7.02 ± 0.08	64.71 ± 0.20	129.52 ± 0.81	0	62.30 ± 1.75
GPP	130 mL dH_2_O	7.90 ± 0.09	67.38 ± 0.66	129.12 ± 1.17	0	62.05 ± 4.08
Pear pulp	320 g pear,	1.20 ± 0.05	17.71 ± 2.25	50.55 ± 3.87	0	33.49 ± 2.49
Pear juice	50 mL dH_2_O	0.98 ± 0.02	17.89 ± 2.45	44.51 ± 2.59	0	29.46 ± 3.31
Persimmon pulp	320 g persimmon,	5.02 ± 1.14	75.79 ± 2.43	81.88 ± 3.64	0	0
Persimmon juice	100 mL dH_2_O	5.55 ± 1.58	85.89 ± 3.33	93.48 ± 3.84	0	0

*dH*_*2*_*O* distilled water, *CTW* Chinese traditional wine, *YPP* yellow pear paste, *GPP* green pear paste

The initial pHs of CTW, YPP, and GPP were pH 4.0, 4.50, and 4.65, respectively, which were then adjusted to a pH of 5.3 using 1 N KOH and HCl. The initial pH of pear fruit and pulp was pH 5.3, and the initial pH of persimmon fruit and pulp was pH 5.6; these were used directly without pH adjustment. The product diagrams of raw materials and pictures of processed fruits and commercial high-sugar-content products are included in Additional file 1: Fig. S2. Detailed information on the recipe as well as the initial concentrations of sugars and sorbitol in the fermentation media is shown in Table 1.

### Strains and growth conditions

*Zymomonas mobilis* subsp*. mobilis* ZM4 (ATCC 31821) (Seo et al. 2005) and *Saccharomyces cerevisiae* BY4743 were used in this study. *Z. mobilis* ZM4 was cultured in Rich Medium (RM: 10 g/L yeast extract, 2 g/L KH_2_PO_4_, with different concentration of glucose or fructose, pH 5.8) at 30 ℃ without shaking as previously described (Yang et al. 2020). The sugars used in RMG5, RMG10, RMF5.5, RMF11, RMG5F5.5, and RMG10F11 were 50 g/L glucose, 100 g/L glucose, 55 g/L fructose, 110 g/L fructose, 50 g/L glucose and 55 g/L fructose, 100 g/L glucose and 110 g/L fructose, respectively. *S. cerevisiae* was cultivated in sterile Yeast Peptone Dextrose (YPD) broth (10 g/L yeast extract, 20 g/L peptone, 20 g/L glucose) at 30 ℃ with shaking at 200 rpm.

### Fermentation

Cell cultures grown as mentioned above to the logarithmic phase were centrifuged at 4000 rpm for 10 min at room temperature and washed once with sterile water. They were then resuspended and added in 40 mL fermentation medium in a 50-mL flask. The fermentation condition was 30 ℃ without shaking with an initial OD _600 nm_ value of 0.15 for *Z. mobilis* and 30 ℃, 200 rpm shaking with an initial OD _600 nm_ value of 0.50 for *S. cerevisiae*. Each experiment was performed in triplicates, and cultures were sampled at different time points post-inoculation to monitor cell growth and concentrations of fructose, glucose, sucrose, ethanol, and sorbitol during fermentation.

### Analytical methods

Cell growth in terms of its optical density at 600 nm was monitored with a UV–visible spectrophotometer UV-1800 (AoYi Instrument Co., Ltd, Shanghai, China). Samples were centrifuged at 12,000 rpm for 2 min, and supernatants were filtered using a 0.45-μm filter. The concentrations of sucrose, glucose, fructose, ethanol, and sorbitol were determined by high-performance liquid chromatography (HPLC, Shimadzu, Japan) equipped with a refractive index detector (RID) and a column of Bio-Rad Aminex HPX-87H (300 × 7.8 mm). The mobile phase was 0.005 M H_2_SO_4_ with a flow rate of 0.5 mL/min, and the temperatures of detector and column were 40 and 60 ℃, respectively. The concentration of sucrose was determined using a flow rate of 0.3 mL/min, and the temperatures of 35 and 18 ℃ for detector and column were used, respectively, to avoid the digestion of sucrose in the hot dilute acid (Duarte-Delgado et al. 2015).

The total consumed sugar (*C*_Total_) was calculated according to the following formulas:

$$C_{{{\text{Total}}}} = \left( {S*0.526 + G} \right) \, + \, \left( {S*0.526 + F} \right).$$

In this equation, “*S*” means sucrose consumed, “*G*” means glucose consumed, and “*F*” means fructose consumed. “0.526” is the theoretical yield from sucrose into glucose and fructose.

When sorbitol was produced in media containing both fructose and glucose, the total sugars consumed for ethanol and other end-products (*C*_Ethanol_) was calculated according to the following formula with the fructose used for sorbitol production subtracted:

$$C_{{{\text{Ethanol}}}} = \, C_{{{\text{Total}}}} - {\text{Sorbitol}}/1.011.$$

“Sorbitol” means the amount of sorbitol produced. “1.011” is the theoretical yield from fructose into sorbitol.

The ethanol yield (*Y*_*E*_) is calculated according to the following formulas (Günan Yücel and Aksu 2015):

$$Y_{E} = \, g \, \max {\text{ ethanol}}/C_{{{\text{Ethanol}}}} .$$

In this equation, “g max ethanol” is the maximum theoretical ethanol produced.

The theoretical ethanol yield (*Y*_*E*_%) is calculated according to the following formula (Demiray et al. 2019):

$$Y_{E} \% = \, \left[ {g \, \max {\text{ ethanol}}/\left( {C_{{{\text{Ethanol}}}} *0.511} \right)} \right] \, *100.$$

### Statistical analysis

Data were analyzed by *t*-tests or one-way ANOVA using the GraphPad Prism statistical software (version 8.0.1). *p* < 0.05 was considered as statistically significant difference.

## Results and discussion

### Fermentation performance of *Z. mobilis* in pure sugars of glucose and fructose

**Fig. 1:**
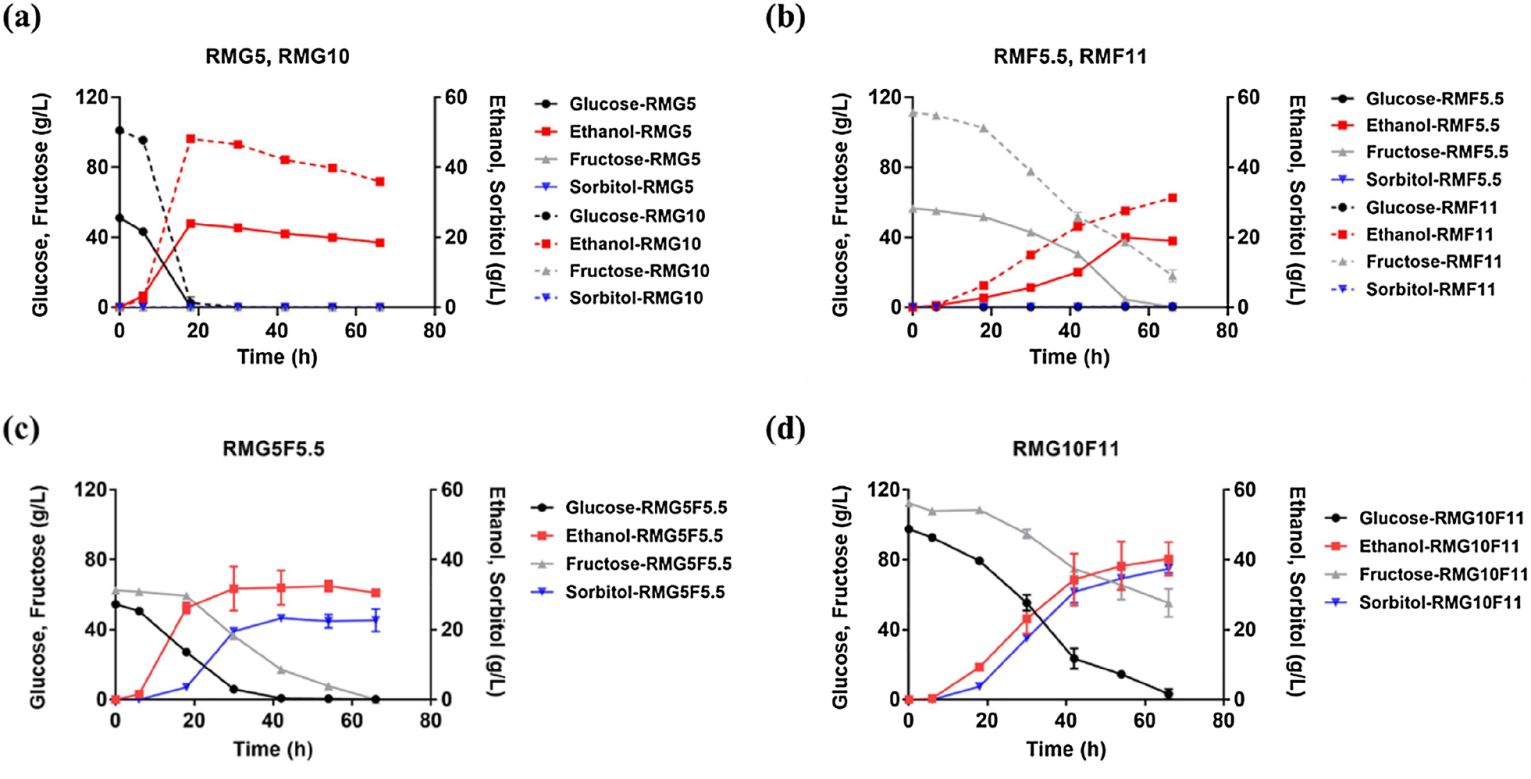
Fermentation performance of *Z. mobilis* in RM medium including the consumption of glucose (black circle) and fructose (grey triangle) as well as the production of ethanol (red square) and sorbitol (blue inverted triangle) in RMG5 and RMG10 (**a**), RMF5.5 and RMF11 (**b**), RMG5F5.5 (**c**), and RMG10F11 (**d**)

*Z. mobilis* was cultured directly in pure sugars of glucose, fructose, as well as mixed sugars of glucose and fructose in different concentrations of RMG5, RMG10, RMF5.5, RMF11, RMG5F5.5, and RMG10F11 to compare sugar utilization as well as ethanol and sorbitol production. Our results demonstrated that glucose is the preferable sugar compared to fructose for *Z. mobilis*, and sorbitol cannot be produced by *Z. mobilis* in monosaccharide medium of glucose or fructose (Fig. 1).

Within 20 h post-inoculation, all glucose up to the highest 100 g used in this study was consumed with an ethanol yield around 0.48 g/g, while it took more than 60 h to utilize 55 g fructose with an ethanol yield around 0.35 g/g (Fig. 1a, b; Table 2). In addition, the presence of fructose in the mixed sugars of glucose and fructose severely impeded the glucose utilization despite that glucose was still consumed first following by a concurrent utilization of glucose and fructose and simultaneous production of ethanol and sorbitol (Fig. 1c, d; Table 2). Sorbitol formation in *Z. mobilis* fermentations is a result of glucose-fructose oxidoreductase (Gfo), which is involved in the complete catalytic cycle of oxidation of glucose to gluconate with concomitant reduction of fructose to sorbitol (Additional file 1: Fig. S1).

**Table 2:** Sugar consumption as well as production of ethanol and sorbitol of *Z. mobilis* in RM media with different concentrations of fructose and glucose

Medium	Glucose consumption (g/L)	Fructose consumption (g/L)	Ethanol production (g/L)	Sorbitol production (g/L)		Ethanol yield (g/g)	Ethanol percent yield (%)	
RMG5	51.19	0	23.90 ± 0.03	0		0.47 ± 0.01	91.39 ± 0.12	
RMG10	101.00	0	48.11 ± 0.98	0		0.48 ± 0.01	93.21 ± 1.90	
RMF5.5	0	56.55	19.90 ± 0.31	0		0.35 ± 0.36	68.87 ± 1.06	
RMF11	0	93.36 ± 3.47	31.33 ± 0.71	0		0.34 ± 0.01	65.70 ± 0.95	
RMG5F5.5	54.33	62.56	33.80 ± 1.36	22.69 ± 0.19		0.36 ± 0.05	70.20 ± 2.13	
RMG10F11	94.25 ± 2.88	57.29 ± 2.08	40.24 ± 1.76	37.41 ± 1.26		0.35 ± 0.01	68.68 ± 2.31	

### Application and comparison of *Z. mobilis* with yeast for sugar reduction in fruits

Sugar utilization capability of *Z. mobilis* in high-sugar-content fruits was then investigated using two fresh fruits of pear and persimmon with minimal processing of adding distilled water to the fruit slurries. Hebei snow pear used in this study contained ca. 30 g/L sorbitol while the concentrations of fructose and glucose were low (ca. 60 g/L) compared to those in Guangxi persimmon, which had no sorbitol detected but contained ca. 160 g/L total sugars of fructose and glucose (Table 1).

**Fig. 2:**
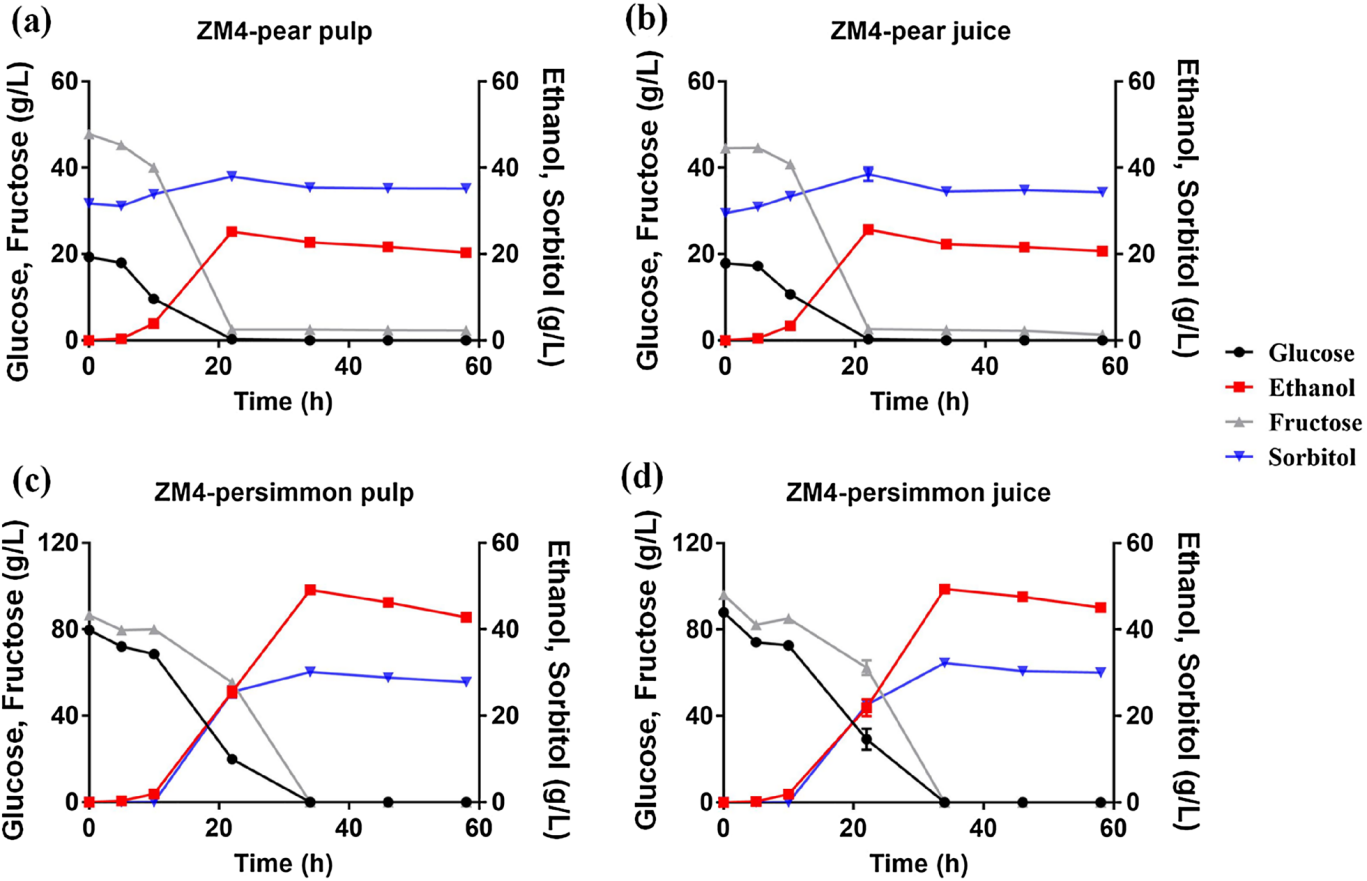
Concentration changes of glucose (black circle), ethanol (red square), fructose (grey triangle), and sorbitol (blue inverted triangle) during fermentation by *Z. mobilis* in pear pulp (**a**), pear juice (**b**), persimmon pulp (**c**), and persimmon juice (**d**), respectively. There was no significant difference for *Z. mobilis* fermentation in both fruit pulp and juice with *T*-test *p*-value greater than 0.05

*Z. mobilis* can utilize all sugars in the fruit juices and pulps with ca. 20 g/L and 40 g/L ethanol produced from pear and persimmon fruits, respectively (Table 3; Fig. 2). The noticeable bubbles observed during fermentation in fruit juices and the pores formed during fermentation using persimmon pulp could be due to the release of carbon dioxide from cell growth and sugar metabolism, which also suggests that *Z. mobilis* can utilize sugars efficiently in the fruit juices and pulps.

**Table 3:** Sugar consumption as well as the production of ethanol and sorbitol of *Z. mobilis* in fruit juice and pulp

	Glucose consumption (g/L)	Fructose consumption (g/L)	Ethanol production (g/L)	Sorbitol production (g/L)	Ethanol yield (g/g)	Ethanol percent yield (%)
Pear pulp	19.30	45.49 ± 0.05	20.31 ± 0.73	3.43 ± 0.56	0.33 ± 0.01	64.75 ± 2.72
Pear juice	17.89	43.20 ± 1.18	20.68 ± 0.24	4.88 ± 0.87	0.37 ± 0.01	71.98 ± 2.32
Persimmon pulp	79.64	86.57	42.79 ± 0.74	27.76 ± 0.11	0.31 ± 0.01	60.36 ± 1.01
Persimmon juice	87.92	96.20	45.09 ± 0.90	30.04 ± 0.45	0.29 ± 0.01	57.15 ± 1.02

Fermentation experiments using pear and persimmon were repeated three and six times, respectively. The R-squared values among different batches of experiments ranging from 0.93 to 0.99 demonstrated the great reproducibility of using *Z. mobilis* for sugar reduction in high-sugar-content fruits (Additional file 1: Fig. S3).

Comparing persimmon with pear, it took *Z. mobilis* more time to consume sugars in persimmon fruit than in pears. This may be due to higher total sugar contents of glucose and fructose in persimmon fruits than those in pear (Tables 1, 3; Fig. 2). In addition, more sorbitol was produced using persimmon fruit than when using pear fruit, reaching a similar total amount around 30 g/L after fermentation and resulting in a lower ethanol yield (Table 3; Fig. 2).

**Fig. 3:**
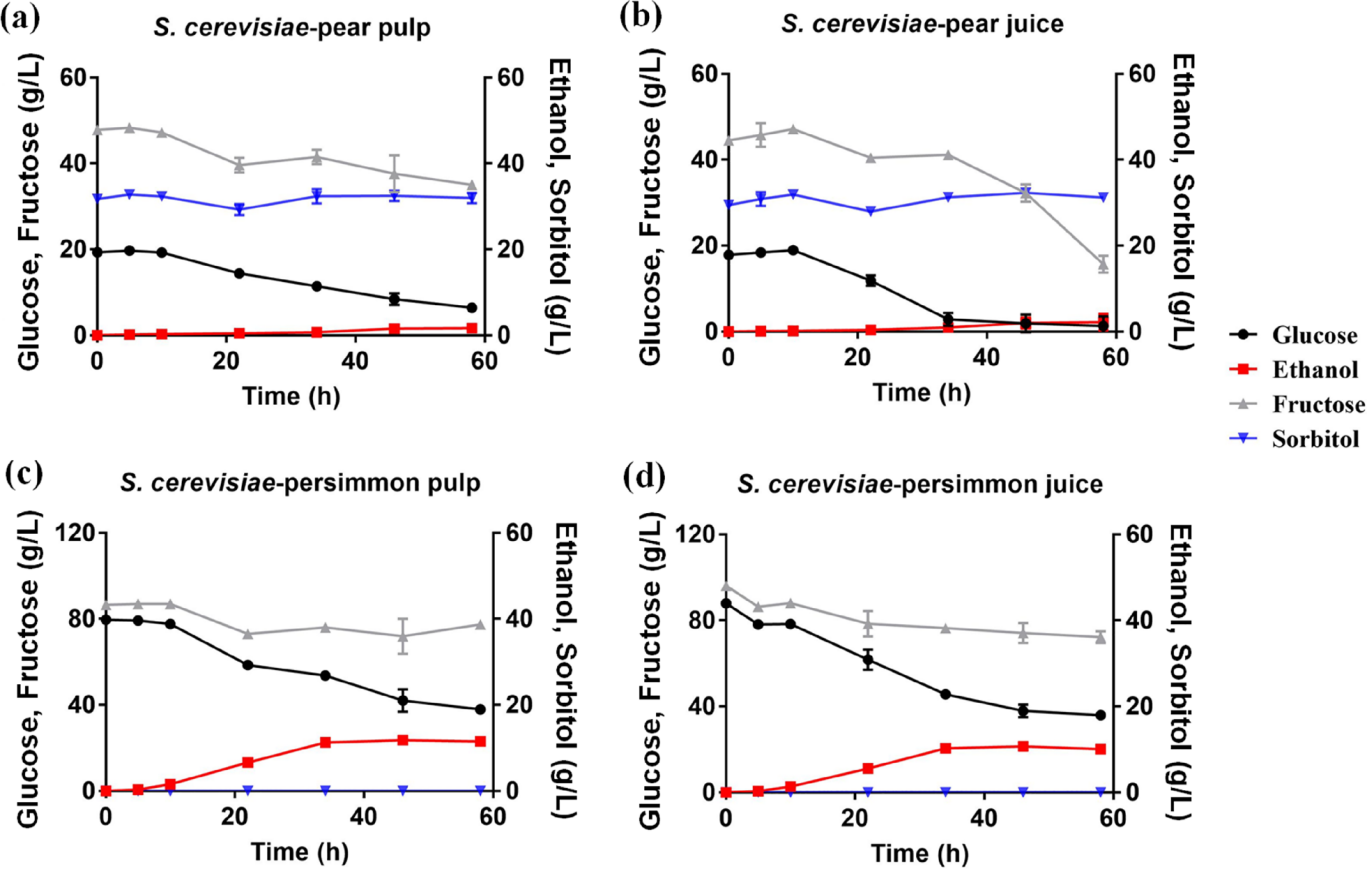
Concentration changes of glucose (black circle), ethanol (red square), fructose (grey triangle), and sorbitol (blue inverted triangle) during fermentation by *S. cerevisiae* in pear pulp (**a**), pear juice (**b**), persimmon pulp (**c**), and persimmon juice (**d**), respectively. There were significant differences for glucose consumption (*p*-value = 0.02) and sorbitol production (*p*-value = 0.008) using pear pulp and pear juice

The fermentation performance of the classical industrial ethanologen yeast was also investigated using pear and persimmon fruits. Our results indicated that although yeast can also utilize glucose and fructose in the fruit juices and pulps, it took a longer time to utilize only part of the sugars in the media and produced a little amount of ethanol and no sorbitol (Fig. 3). There was more than half (35.04 ± 0.40 g/L) and one-third (15.75 ± 1.97 g/L) fructose left after 60 h fermentation by *S. cerevisiae* in the pear pulp and juice, respectively (Fig. 3a, b). The ethanol yield using persimmon by *S. cerevisiae* was higher than that of using pear, which was up to 0.23 ± 0.01 g/g (Fig. 3c, d). However, the sugars were consumed much slower by yeast than *Z. mobilis* (Fig. 2) with a significant amount of glucose left in the media and only a small amount of fructose being utilized 60 h post-inoculation (Fig. 3). Therefore, *Z. mobilis* was more suitable than *S. cerevisiae* BY4743 to reduce sugars quickly and efficiently in pear and persimmon fruits with minimal processing.

### Application and comparison of *Z. mobilis* with yeast for sugar reduction in commercial high-sugar products

We further examined the sugar reduction capabilities of *Z. mobilis* in two commercial products: yellow pear paste (YPP) and green pear paste (GPP). The major components of sugars glucose and fructose as well as sorbitol in the pear pastes (PPs) are similar to those in the pear fruit containing significant amounts of sorbitol (> 60 g/L) and onefold more fructose (ca. 130 g/L) than glucose (Table 1). The major difference between GPP and YPP is the medium color of GPP was lighter than that of YPP (Additional file 1: Fig. S2), and the sucrose and glucose concentrations in GPP were slightly higher than those in YPP (Table 1).

**Fig. 4:**
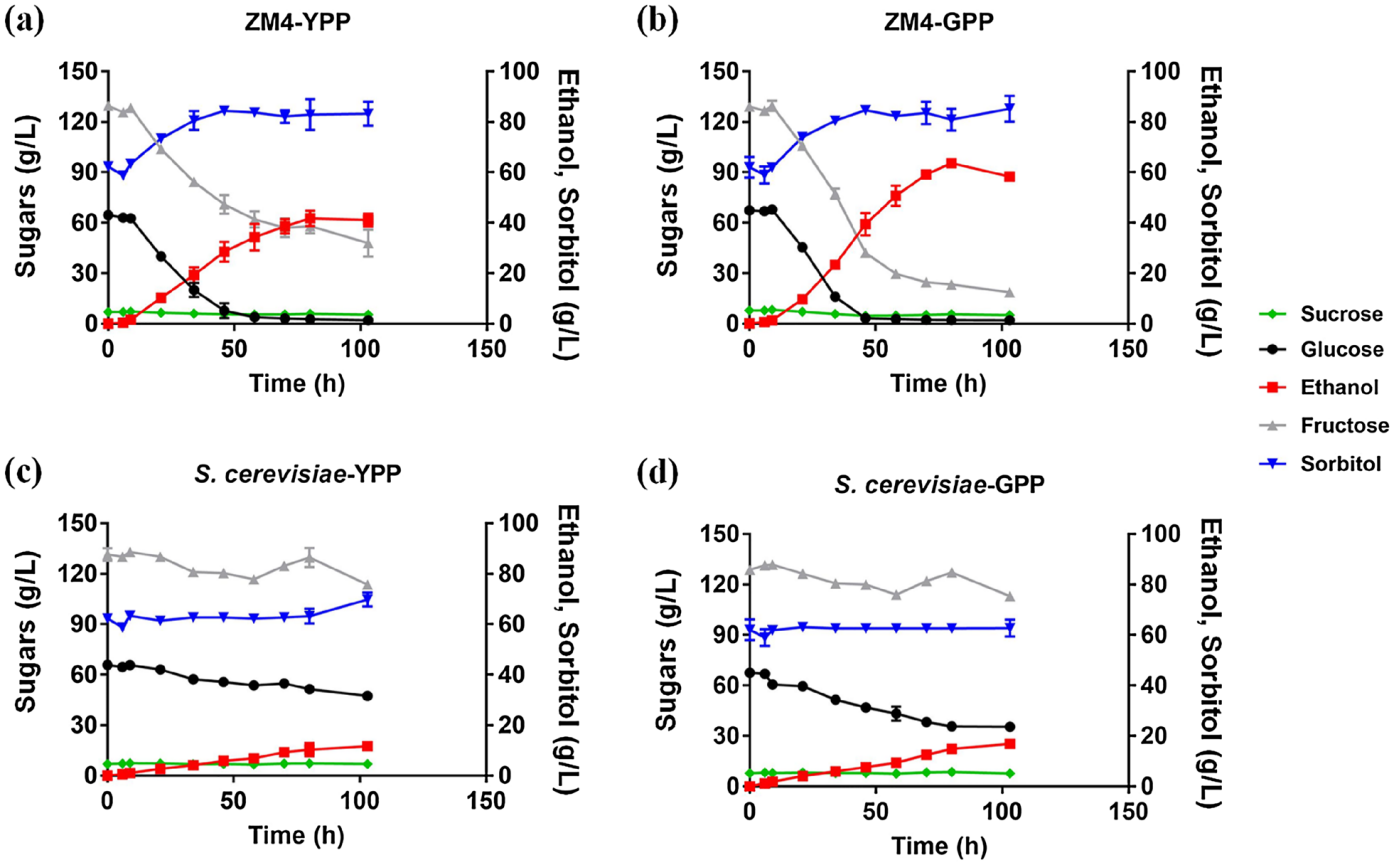
Concentration changes of sucrose (green diamond), glucose (black circle), ethanol (red square), fructose (grey triangle), and sorbitol (blue inverted triangle) during fermentation in pear pastes of yellow pear paste (YPP, **a**) and green pear paste (GPP, **b**) by *Z. mobilis* as well as those by *S. cerevisiae* in YPP (**c**) and GPP (**d**), respectively. There were significant differences for fructose consumption and ethanol production in GPP and YPP for *Z. mobilis*, as well as glucose consumption and ethanol production in GPP and YPP for *S. cerevisiae* with a T-test *p*-value ≤ 0.01

*Z. mobilis* can consume all glucose (> 60 g/L) within 50 h post-inoculation and most fructose (> 80 g/L) with similar amounts of sorbitol produced (> 20 g/L) in the PPs (Table 4; Fig. 4a, b). However, *Z. mobilis* consumed fructose slower in YPP than that in GPP with more than 30 g/L fructose left and 40% less ethanol produced correspondingly. The final alcohol concentrations in YPP and GPP were 41.10 ± 2.51 g/L and 58.11 ± 0.60 g/L with ethanol percent yields of 64.50 ± 1.12% and 73.06 ± 1.86%, respectively (Table 4; Fig. 4a, b), which is consistent with a previous study finding that ethanol yield of *Z. mobilis* SBE15 in four sugar beet substrates were reduced to 73 ~ 79% due to sorbitol formation (Park and Baratti 1991).

**Table 4:** Sugar consumption and ethanol conversion rate of *Z. mobilis* in pear paste

	Sucrose consumption (g/L)	Glucose consumption (g/L)	Fructose consumption (g/L)	Ethanol production (g/L)	Sorbitol production (g/L)	Ethanol yield (g/g)	Ethanol percent yield (%)
YPP	1.61 ± 0.15	63.46 ± 0.21	82.43 ± 2.82	41.10 ± 2.51	20.90 ± 1.50	0.33 ± 0.02	64.50 ± 1.12
GPP	2.72 ± 0.19	66.79 ± 0.71	111.88 ± 2.66	58.11 ± 0.60	23.18 ± 1.22	0.37 ± 0.01	73.06 ± 1.86

*YPP* yellow pear paste, *GPP* green pear paste

Similarly, the fermentation performance of yeast *S. cerevisiae* in these two pear pastes of YPP and GPP was examined (Fig. 4c, d). The results indicated that yeast also performed better in GPP than in YPP. Similar to the fermentation performance using fresh pear fruits, *S. cerevisiae* consumed sugars in the PPs slowly with most sugars left and little ethanol produced. For example, it only consumed about 32 g glucose and 16 g fructose with less than 17 g ethanol produced after fermentation completed 100 h post-inoculation; this was less than one-third of that produced by *Z. mobilis* (Table 4; Fig. 4).

### Application and comparison of *Z. mobilis* with yeast for sugar reduction in commercial high-sugar alcoholic products

Chinese traditional wines are fermented alcoholic beverages brewed directly from different combinations of grains such as millet, rice, and wheat with an alcohol content around 10 ~ 20%. We first measured the concentrations of ethanol and major sugars in the Chinese traditional wine (CTW), and the result exhibited that the CTW we used in this study contained more than 100 g/L ethanol. The majority of sugars in the CTW was glucose (> 120 g/L) with ca. 20 g/L sucrose and a small amount of fructose around 2 g/L (Table 1).

**Fig. 5:**
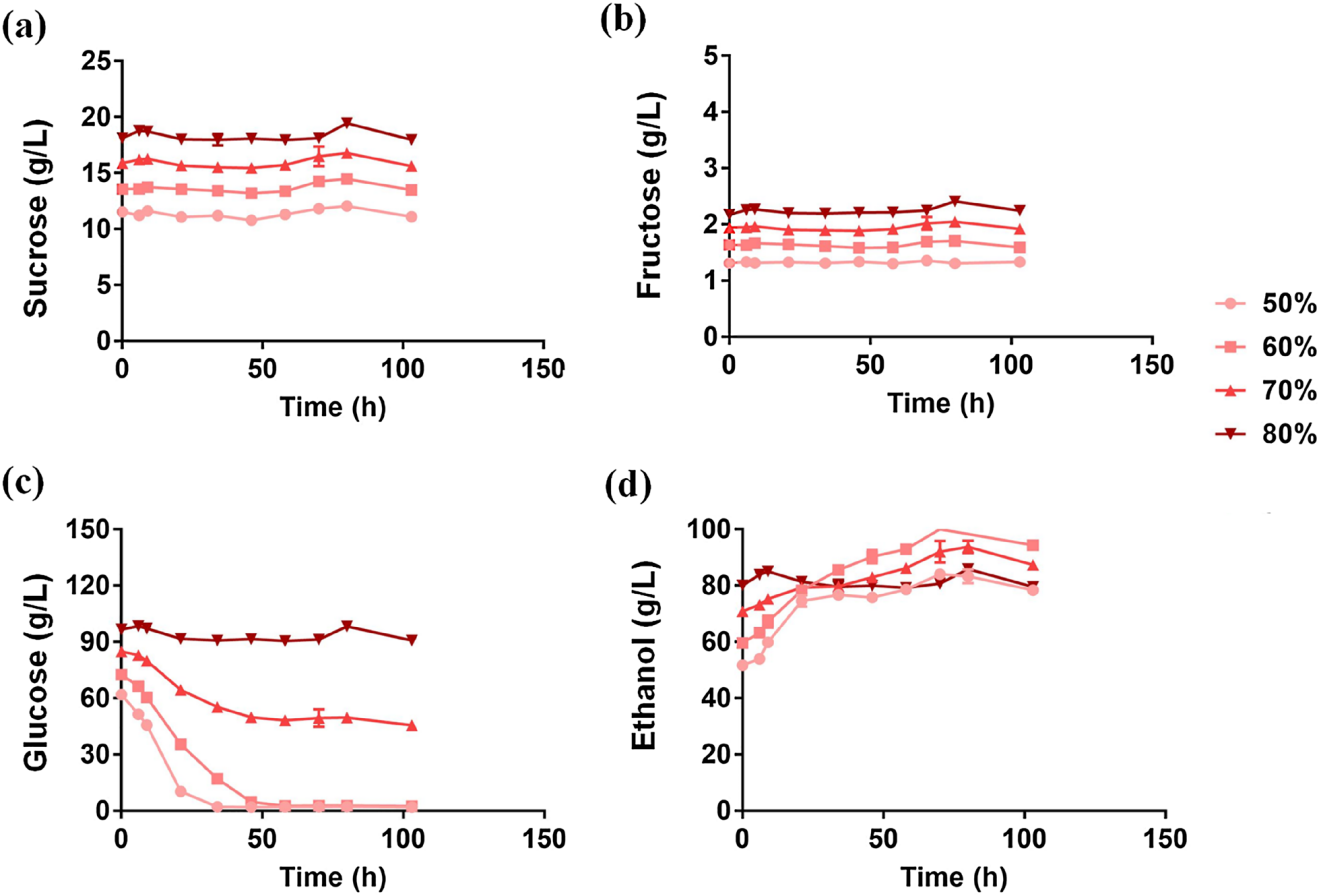
Concentration changes of sucrose (**a**), fructose (**b**), glucose (**c**), and ethanol (**d**) during fermentation by *Z. mobilis* in Chinese traditional wine (CTW) with the dilution rates of 50% (circle), 60% (square), 70% (triangle), and 80% (inverted triangle), respectively. There were significant differences for glucose consumption (*p-*value < 0.0001) and ethanol production (*p-*value = 0.035) in 50%, 60%, 70%, and 80% CTW using one-way ANOVA

Considering that the growth of *Z. mobilis* will be inhibited when ethanol concentration is above 10%, we diluted the CTW with distilled water to different final concentrations of 50, 60, 70, and 80% CTW. *Z. mobilis* utilized all glucose in 50% and 60% CTW within 2 days with 26.67 ± 0.03 and 34.75 ± 1.75 g/L ethanol produced, resulting in a final ethanol concentration of 84.06 ± 0.51 g/L and 100.97 ± 1.70 g/L, respectively (Tables 1, 5; Fig. 5). *Z. mobilis* utilized half of the glucose in 70% CTW, but only 5 g glucose was consumed in 80% CTW. These results indicated that the optimal concentration for sugar reduction in CTW by *Z. mobilis* is 60%, which had the lowest dilution but highest ethanol titer and yield (Tables 1, 5; Fig. 5).

**Table 5:** Substrate sugars consumption and ethanol conversion rate of *Z. mobilis* in Chinese traditional wine (CTW)

CTW	Sucrose consumption (g/L)	Glucose consumption (g/L)	Fructose consumption (g/L)	Ethanol production (g/L)	Ethanol yield (g/g)	Ethanol percent yield (%)
50%	0.45 ± 0.14	60.24 ± 0.18	0.37 ± 0.71	26.67 ± 0.03	0.44 ± 0.01	86.16 ± 1.57
60%	0.09 ± 0.20	70.08 ± 0.73	0.10 ± 0.03	34.75 ± 1.75	0.50 ± 0.03	96.97 ± 2.21
70%	0.27 ± 0.12	39.39 ± 2.41	0.17 ± 0.05	16.54 ± 0.45	0.42 ± 0.04	81.83 ± 2.35
80%	0.15 ± 0.24	5.27 ± 1.16	0.15 ± 0.30	0.55 ± 0.47	0.10 ± 0.06	19.86 ± 1.19

No sorbitol detected

*S. cerevisiae*, however, was unable to consume sugars in CTW in these concentrations (Additional file 1: Fig. S4), even when supplemented by YPD^−^ solution into CTW to supply an exogenous nitrogen source for *S. cerevisiae*. Although *S. cerevisiae* BY4743 can tolerate 8% (v/v) ethanol (Nilambari and Jadhav 2010), ethanol concentrations in 50, 60, 70 and 80% CTW were 51.68 ± 0.13, 59.66 ± 0.90, 70.87 ± 0.13, and 80.08 ± 0.24 g/L, respectively (Table 1). Therefore, inhibitors in CTW including ethanol could impede the growth of *S. cerevisiae* in CTW.

Differing from conditions where fruits and pear pastes were used as the materials, no sorbitol was detected in CTW when fermented by *Z. mobilis.* This could be due to the low fructose concentration (less than 2 g/L) in CTW. Although CTW contains sucrose (Table 1), the SacC enzyme of *Z. mobilis* that hydrolyzes sucrose to fructose and glucose could be inhibited in a high ethanol environment, resulting in little fructose generation and sorbitol production.

CTW may also be served as medicine, which can be the case for the one used in this study. High-sugar content in these wines could cause excessive intake of sugar, which is not suitable for patients sensitive to sugar such as those with cancers or diabetes. Our study thus provides an alternative strategy to reduce sugar in high-sugar-content wines, including medicinal alcoholic drinks. Despite *Z. mobilis* being more advantageous than yeast for reducing sugars in materials of high-sugar-content fruits and commercial products used in this study, we only tested limited strains of *Z. mobilis* ZM4 and *S. cerevisiae* BY4743 under limited conditions. It is possible that other microorganisms, such as other yeast strains or other probiotic microorganisms can fulfill a similar role to *Z. mobilis* ZM4 tested in this study. In addition, although our study demonstrated that *Z. mobilis* ZM4 can reduce sugars to ethanol for diverse high-sugar-content fruits and commercial products, the complete metabolic profiles after fermentation should be investigated in the future including those that could come from the materials we used (e.g., polyphenols and minerals).

## Conclusions

The potential of applying the bacterial ethanologen *Z. mobilis* for sugar reduction in high-sugar-content fruits and commercial products was evaluated and compared with the classical ethanologen yeast *S. cerevisiae* in this study. Our results demonstrated that *Z. mobilis* performed better than the yeast in high-sugar-content fruits and commercial products used in this study with a fast and efficient sugar utilization and ethanol production. In addition, the whole process is simple and economic-only requiring pH adjustment and appropriate dilution using water-which can easily be scaled up for commercial applications. Considering the excellent capability of *Z. mobilis* to produce sorbitol and levan-type prebiotics as well as its unique characteristics of high ethanol yield in high sugar and ethanol environments given limited nutrient requirements for efficient fermentation demonstrated in this study, more efforts should be spent to utilize this GRAS probiotic strain for its broad applications in food industry.

### Supplementary Information

**Additional file 1: Fig. S1.** Metabolic pathways of *Z. mobilis*. **Fig. S2.** Original and processed materials used in this study. **Fig. S3.** Correlation analysis of fermentation results by *Z. mobilis* in pear and persimmon pulp and juice. **Fig. S4.** Concentration changes during fermentation by *S. cerevisiae* in Chinese traditional wine (CTW) with the dilution rates.

## Data Availability

All data generated or analyzed during this study are included in the manuscript.

## Abbreviations

Adhs: Alcohol dehydrogenases
CTW: Chinese traditional wine
ED: Entner–Doudoroff
EMP: Embden–Meyerhof–Parnas
Gfo: Glucose-fructose oxidoreductase
GPP: Green pear paste
GRAS: Generally regarded as safe status
Pdc: Pyruvate decarboxylase
PPs: Pear pastes
RID: Refractive index detector
RM: Rich medium
YPD: Yeast peptone dextrose
YPP: Yellow pear paste
