# Supplementary material for: Revitalizing the ethanologenic bacterium Zymomonas mobilis for sugar reduction in high-sugar-content fruits and commercial products
Source: Bioresour Bioprocess. 2021 Dec 2;8(1):119. doi: 10.1186/s40643-021-00467-2 (PMC8637514; doi:10.1186/s40643-021-00467-2)
Supplement: Supplementary file 1 — Additional file 1: Fig. S1. Metabolic pathways of Z. mobilis. Fig. S2. Original and processed materials used in this study. Fig. S3. Correlation analysis of fermentation results by Z. mobilis in pear and persimmon pulp and juice. Fig. S4. Concentration changes during fermentation by S. cerevisiae in Chinese traditional wine (CTW) with the dilution rates. [file 40643_2021_467_MOESM1_ESM.pdf]

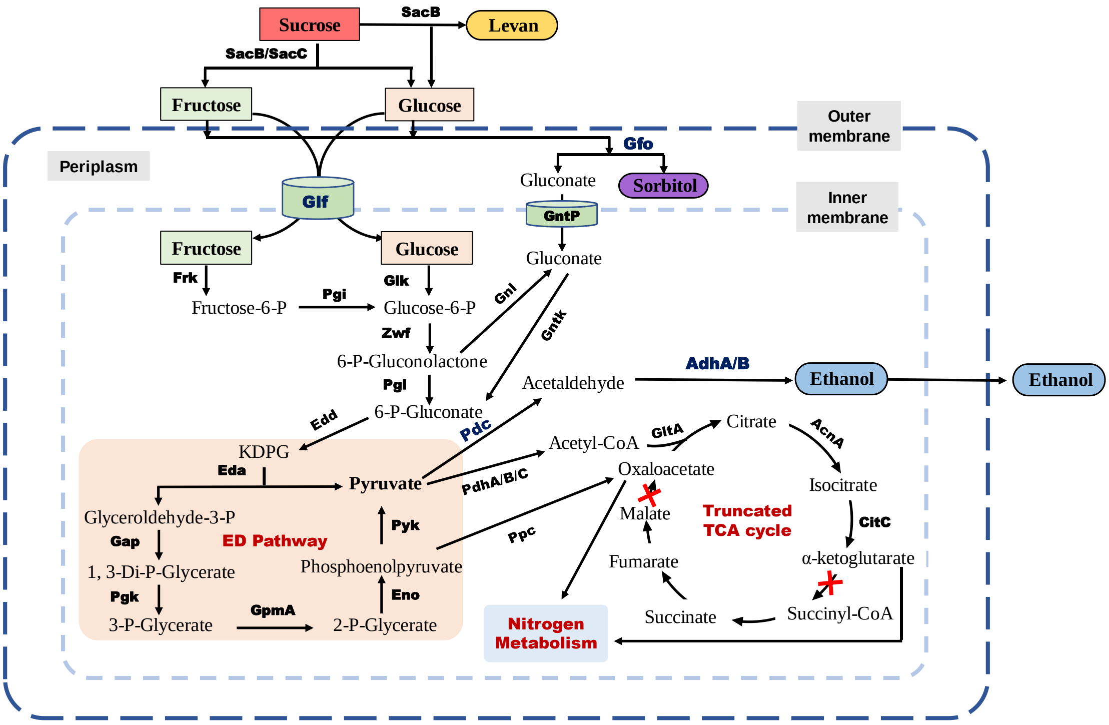

Fig. S1. Metabolic pathways of *Z. mobilis*. Gfo: glucose-fructose oxidoreductase, Glf: glucose facilitator, and KDPG: 2-Keto-3-deoxy-6-P-Gluconate.

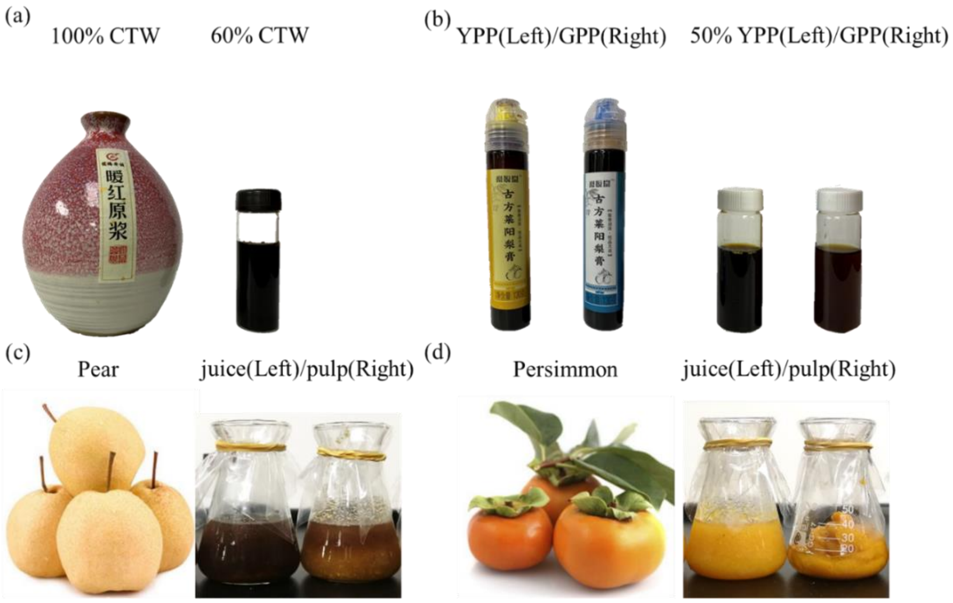

Fig. S2. Original and processed materials of traditional high-sugar-content commercial products of Chinese traditional wine (**a**), and two kinds of pear pastes (PP) of yellow pear paste (YPP) and green pear paste (GPP) (**b**), as well as juice and pulp of pear (**c**), and juice and pulp of persimmon (**d**) used in this study.

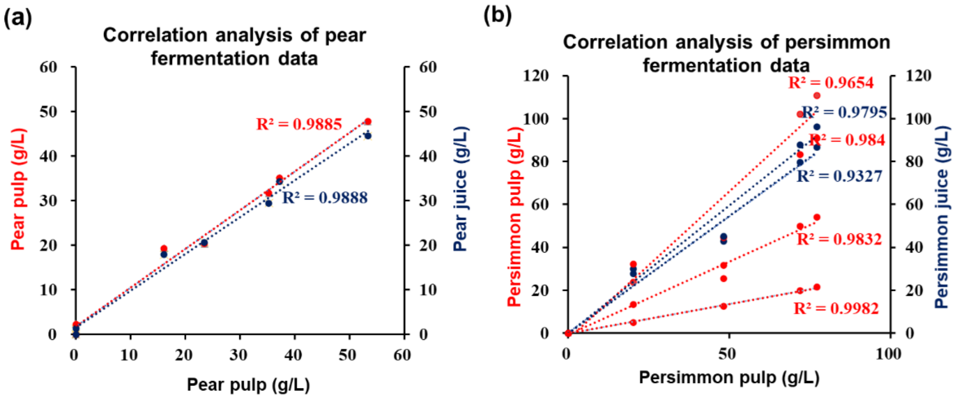

Fig. S3. Correlation analysis of fermentation results by *Z. mobilis* in pear and persimmon pulp and juice. Data comparison of glucose, fructose, ethanol, and sorbitol in two groups of pear pulp and one group of pear juice during fermenting in pear (**a**); Data comparison of glucose, fructose, ethanol, and sorbitol in persimmon pulp, 1/2 persimmon pulp, 1/4 persimmon pulp and persimmon juice during fermenting in persimmon (**b**).

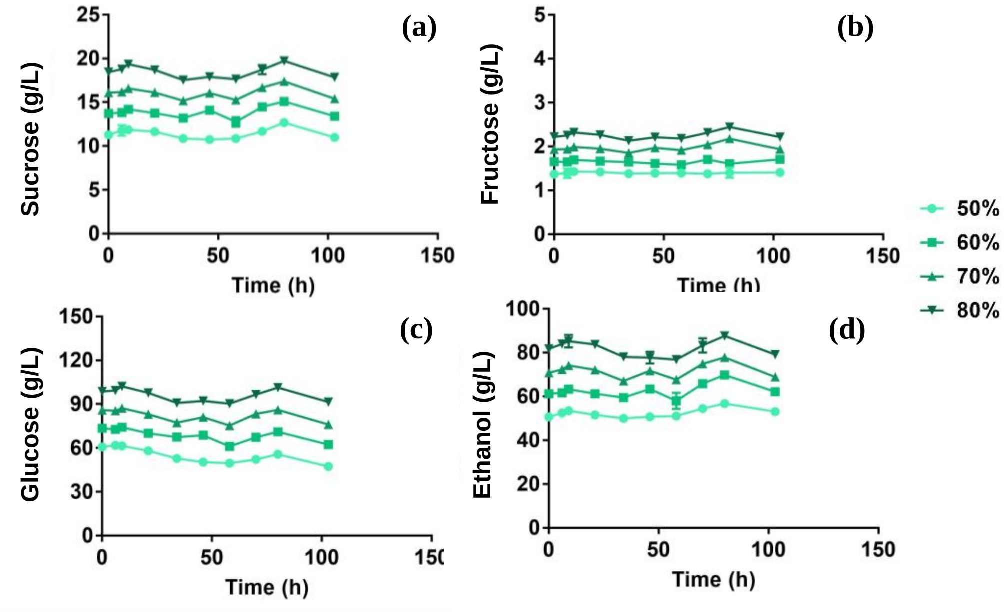

Fig. S4. Concentration changes of sucrose (**a**), fructose (**b**), glucose (**c**), and ethanol (**d**) during fermentation by *S. cerevisiae* in Chinese traditional wine (CTW) with the dilution rates of 50% (●), 60% (■), 70% (▲), and 80% (▼), respectively.
